# A Case of Vesical Retention Cured by Persistent Catheterism

**Published:** 1857-03

**Authors:** Wm. G. Meacham

**Affiliations:** Warsaw, Wyoming Co., N. Y.


ART. IV.—*A Case of Vesical Retention cured by Persistent Catheterism.* By WM. G. MEACHAM, Warsaw, Wyoming Co., N. Y.

David Martin, æt. —, farmer, in April last, applied for medical aid. The history of his case, as given by himself, is as follows:

Ever since the period of his childhood, he had suffered more or less from dysuria. For several years he had also been afflicted with what he termed the "spinal complaint," that is to say, he had had an incessant pain in his loins. About nine years since he had an apoplectic seizure, terminating in incomplete hemiplegia, but gradually recovered from the paralysis, the facial muscles, however, being left somewhat stiff and contracted. Two years or more ago he was attacked with vesical retention, and ever since has labored, to a greater or less degree, under that functional derangement, the retention at times assuming a paroxysmal violence. It was a return of this exacerbation of severity more than usually intense and persistent, that brought me to his bedside.

I found, on examination, a tense, roundish tumor in the hypogastrium, nearly reaching the midway point between the symphysis pubis and the umbilicus, dull on percussion, and painful on pressure and bodily motion — evidently the urinary bladder in a state of distension. The existing condition of things at once indicated the propriety of catheterization, and it was accordingly executed without delay. Three pints, or thereabouts, of urine, were in this way removed, necessarily occasioning the collapse of the tumor, and a sensible diminution in the amount of tensive pain. It was found necessary to repeat the operation daily for two or more weeks, at the end of which time the difficulty was so far overcome that the patient could discharge the contents of the bladder without surgical aid.

The previous history of the case, the patient's age, and the success of the method adopted for relief, unitedly led me to the belief that this was a case of vesical paralysis, induced, partially at least, by continued over-distention with urinous accumulation. For the past two years the patient had frequently, perhaps habitually, suffered his bladder to be unduly taxed, but, as the retention had never been complete, (nature wisely establishing a *stillicidium*, by which that portion of the secretion which was over and above the retentive capacity of the recepticle, overflowed, as it were,) and his suffering, consequently, never intolerable, he had neglected to seek medical advice. He probably regarded his malady as a comparatively insignificant one, and was wholly unconscious of the great danger of permitting the retention to continue. For a long time he had found it necessary to resort to manipulation of the hypogastrium and to active voluntary efforts at straining, in order to relieve the *vesica urinaria* of its surplus of contents. The organ, having undergone as long a continuance of repletion, having been frequently, perchance continually, filled to its normal capacity, and not infrequently crammed to a distended capacity, necessarily must have lost its tonicity and muscular contractility, and thus the chief operative agent in the expulsion of its contents been manacled. That incessant muscular tension of this organ debilitates and will eventually destroy the contractile power of its fibres, is a truth no less corroborated by analogy in the case of other muscular strictures, than sanctioned by philosophy and common sense. But it is needless to linger with the proof of a pathological statement so generally accredited as this.

Indeed, it is not requisite to the induction of vesical palsy that the urine should collect beyond, or even up to, the normal capacity of its containing organ, for if the bladder be allowed to remain incessantly charged to any considerable degree, even short of distention, for a length of time, the paralysis will as surely, though not as speedily, ensue. The reason is obvious. Nature's wise design is contravened, and all interference with her regulations brings in its train a penalty. Nature's manifest intention is, that the bladder, equally with every other member of the body, shall have its times of comparative, if not absolute, rest, and, to secure its accomplishment, she has so constructed that viscus, and associated it with the great nervous centres, that whenever a certain quantity of urine has been deposited in its cavity, a quantity which the organ can retain without occasioning tensive or other unpleasant sensations, all further additions so impress the terminal branches of the sensitive nerves distributed throughout the mucous membrane, that an influence is transmitted to the medulla spinalis and the brain, which nervous centres return along the motor filaments to the *detrusor urinæ*, the one the energy of the will, the other, its reflex power, by which that muscle's contractility is set in action, and thus the urine excreted. The diaphragm and abdominal muscles, it must be remembered, however, are also called into play by the will in ordinary urination. This constitution of things, it is evident, necessitates a periodical emptying of the bladder, and, as a consequence, ensures a succeeding interval of comparative rest. Though upon a superficial view it may seem paradoxical to apply the term rest, to the cessation of the full state of this organ, even when short of distention, a closer investigation will, I doubt not, disabuse the mind of any such erroneous impression. To comprehend the appropriateness of the term, it must be borne in mind that a filled condition of this viscus is not its normal, or at least its *continuing* normal state; and that the retaining of a full supply of urine, even this side of actual distention, involves both mechanical resistance and muscular exercise which require a proportionate amount of rest. The mechanical resistance referred to is the simple force of cohesion in the parietes of the bladder, while the muscular exercise is that comparatively trifling degree of contractility excited in the *detrusor urinæ*, by even the smallest volume of urinary contents, but which is increased in direct ratio with the augmentation of urine. Though this contracting force be not considerable, as long as the bulk of renal secretion does not exceed the healthy standard capacity of the bladder, yet an incessant and long-continued exercise of it must inevitably seriously debilitate the muscle. Hence the correctness of the term rest, as applied to the space of time immediately succeeding the act of urination. It is of course rest simply in a relative sense.

The retention in my patient's case, I have no hesitation in saying, was of this character, primarily being due to the combined influences of irritation of the neck of the bladder, and muscular and nervous debility attendant upon advanced years; and secondarily, to weakness of the organ occasioned by its own distention, the disease multiplying itself in a sort of geometrical ratio. The irritation at the neck, in this instance, may have been induced either by a vesical calculus, by gravel, by acrid urine, or by contiguous sympathy with an inflamed prostate, so frequently the portion of advanced life. The retention, moreover, served to re-create itself, not simply through the debilitating influence of over-distention, but also, and perhaps to an equally serious degree, by the exhausting agency exerted by the decomposed urine upon the nervous excitability of the vesical mucous membrane, thereby rendering the bladder so insensible to the presence of excess or acridity of contents that no contractions of its muscular fibres, or, at least, but an insufficiency for the expulsion of the urine, could be elicited.

Though I report this case of retention as cured by persistent catheterism, I wish it to be received as such in a restricted sense, simply as a cure of a paroxysm of retention, and not as a radical cure of my patient's malady. The original causative agencies of the obstruction are yet, I doubt not, in existence, and will, in due course of time, again manifest themselves. If the primitive causation be, as I strongly suspect, lithiasis, my cure is of course but temporary, and nothing but surgical interference for the removal of the calculus, and constitutional remedies addressed to the peculiar diathesis, can avail for a permanent cure. All that is claimed for catheterism as a remedy in these cases, is, that it prevents the fatal occurrence of rupture of the bladder, and paralysis from distention and long-continued irritation by acrid urine, and, by removing a burden, as it were, from the viscus, enables it to regain strength and contract efficiently upon its contents.

There are, indeed, cases in which catheterization may prove a means even of radical cure — those in which the original cause was of transient continuance, as spasm of the neck, or in truth any case in which the primary exciting agency has ceased to act; while the loss of vesical sensibility and contractility, with its consequence, retention, continues. In such instances the retention is evidently its own continuer. Let the distention be removed, and the relieved organ, gathering strength and energy, will remove the retention. Nature is ever willing to aid herself, and when kindly assisted, gently jogged forward by the hand of the skilled physician, is often competent to her relief and preservation. In all cases, however, catheterization is, at times, highly serviceable, and in many, indispensably necessary.

